# Supplementary material for: Microglial amyloid beta clearance is driven by PIEZO1 channels
Source: J Neuroinflammation. 2022 Jun 15;19:147. doi: 10.1186/s12974-022-02486-y (PMC9199162; doi:10.1186/s12974-022-02486-y)
Supplement: Supplementary file 1 — Additional file 1: Fig. S1. PIEZO1 and PIEZO2 expression in human and mouse RNA‑seq dataset and staining controls for hiMGL immunostaining. Fig. S2. Activation of PIEZO1 orchestrates immune response of human iMGLs. Fig. S3. No differences in PIEZO1 expression in bulk brain tissue between WT and 5xFAD mice. Fig. S4. PIEZO1, microglia, astrocyte and Aβ plaque stainings in 5xFAD hippocampi. Fig. S5. PIEZO1 gene expression in published AD‑related RNA datasets by our analysis. Fig. S6. Characterization of MAD1 and MAD8 iPSC lines. Fig. S7. A diagram visualizing author contribution. Fig. S8. A graphical abstract summarizing the main finding of the paper. Table S1. A correlation data for Piezo1 and the DAM signature genes in microglial subpopulations in Keren-Shaul et al. 2017 dataset [41] (GSE98969). Table S2. A correlation data for Piezo1and the DAM signature genes in microglial subpopulations in Zhou et al. 2021 dataset [42] (GSE140511). Table S3. Differentially expressed genes (DEGs) specific for m1‑subcluster in snRNA Grubman etal. 2019 dataset [40] (GSE138852). [file 12974_2022_2486_MOESM1_ESM.docx]

## Additional file 1: Supplementary figures and tables.

# Title: Microglial amyloid beta clearance is driven by PIEZO1 channels

**Authors:** Henna Jäntti^1^†, Valeriia Sitnikova^1^†, Yevheniia Ishchenko^1,2^†, Anastasia Shakirzyanova^1^, Luca Giudice^1,3^, Irene F Ugidos^1^, Mireia Gómez-Budia^1^, Nea Korvenlaita^1^, Sohvi Ohtonen^1^, Irina Belaya^1^, Feroze Fazaludeen^1^, Nikita Mikhailov^1^, Maria Gotkiewicz^1^, Kirsi Ketola^4^, Šárka Lehtonen^1,5^, Jari Koistinaho^1,5^, Katja M Kanninen^1^, Damian Hernández^6,^ Alice Pébay^6,7^, Rosalba Giugno^3^, Paula Korhonen^1^, Rashid Giniatullin^1^, Tarja Malm^1^*


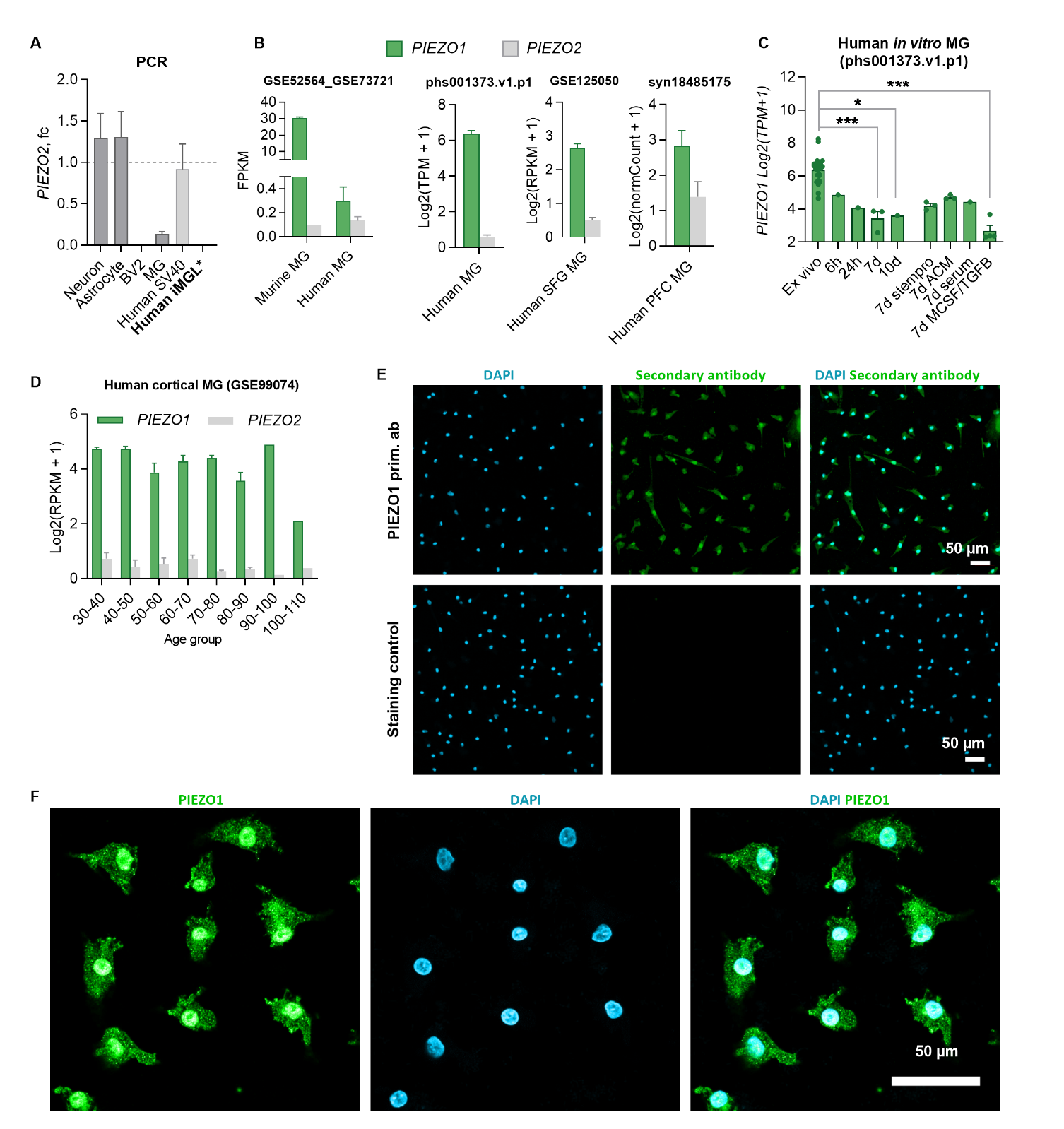
 **Fig. S1. *PIEZO1* and *PIEZO2* expression in human and mouse RNA-seq datasets and staining controls for hiMGL immunostaining. A** *Piezo2* gene expression in murine trigeminal neurons (Neuron), astrocytes (Astro), microglia (MG) and microglial cell line (BV2); and in human microglial cell line (SV40) and iPSC-derived microglia (iMGL) analyzed by RT-qPCR (N=3-4). **B** *PIEZO1* and *PIEZO2* gene expression in microglia (MG) isolated from mouse (GSE52564) and human (GSE73721) brain, human neurosurgical brain tissue (phs001373.v1.p1), human postmortem superior frontal gyrus (SFG; GSE125050), and prefrontal cortex (PFC; syn18485175). **C** *PIEZO1* gene expression in RNA-seq data (phs001373.v1.p1) for *ex vivo* human microglia and cells cultured *in vitro* for up to ten days in medium supplemented with Stempro supplements, astrocyte conditioned medium (ACM) or with human serum, or MCSF and TGFB that are commonly used *in vitro* to support microglial identity. **D** *PIEZO1* and *PIEZO2* levels in superior parietal cortex at different ages (GSE99074). Data obtained for A-D from <http://www.brainrnaseq.org/>. **E** Immunostaining controls and **F** images for figure separated for DAPI (nuclei, blue) and PIEZO1 (green) antibody channels of *in vitro* iMGLs. Unpaired t-test ***p < 0.001, **p < 0.01, *p < 0.05; All data repeated in n experiments each with 3 replicates. Data as mean ± SEM. Exp=experiment; FPKM, Fragments Per Kilobase of transcript per Million; TPM, Transcripts Per Kilobase Million; RPKM Reads Per Kilobase Million.


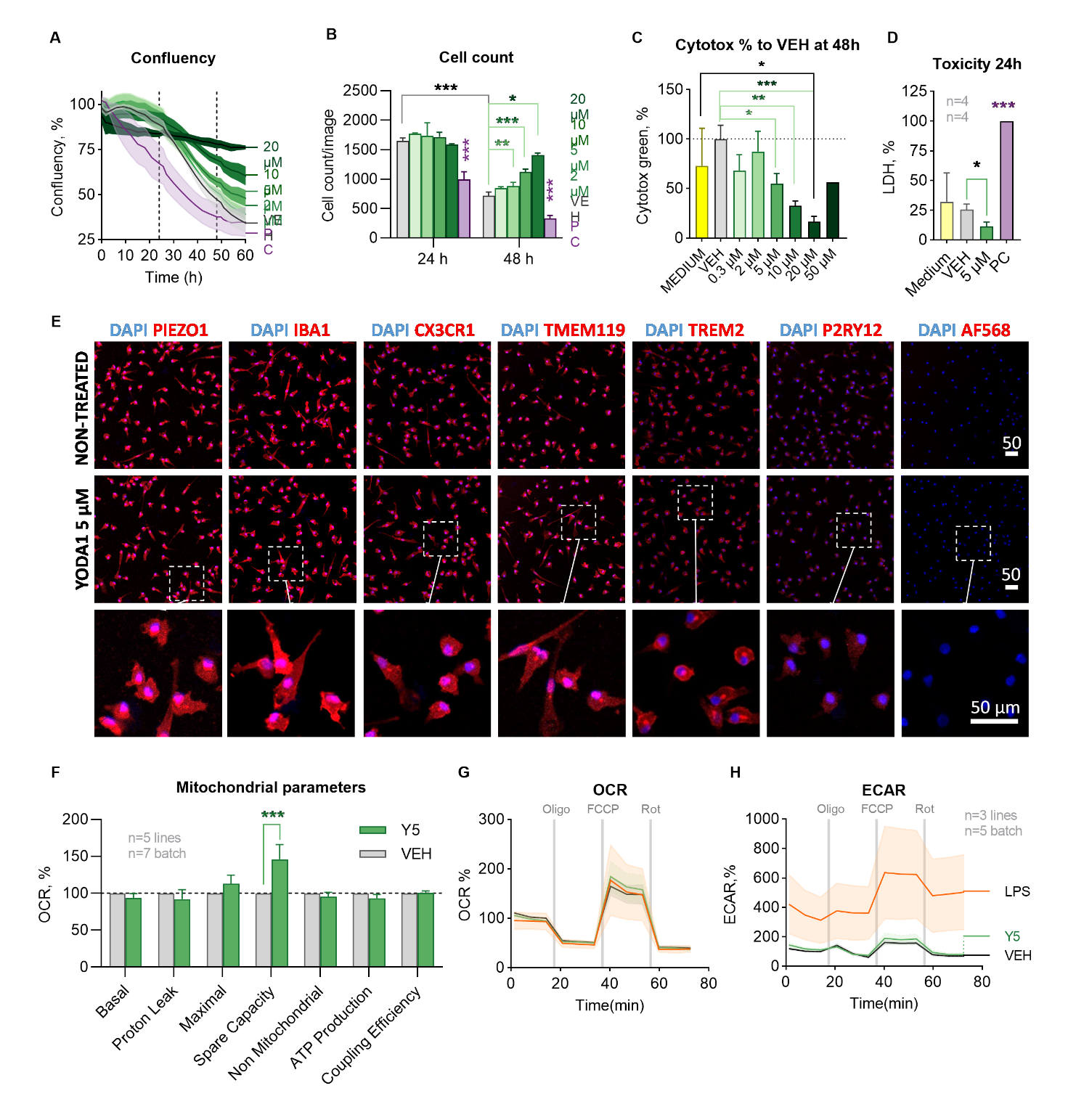
**Fig. S2. Activation of PIEZO1 orchestrates immune response of human iMGLs**. **A** Confluency of iMGLs in cytotox green assay over time (n=3). **B** Cell count at 24 h and 48 h (n=2 wells). **C** Quantification of cytotox green per confluence at 48 h show no differences for medium control, vehicle, 0.3 µM Yoda1 (Y0.3), 2 µM (Y2) Yoda1 (n=2) nor for 50 µM Yoda (n=1). Normalized to vehicle. **D** Lactate dehydrogenase (LDH) toxicity assay from iMGL cell culture medium at 24 h. N=2 for medium, others N=4 in n=4. **E** Representative images of human iMGLs immunostained after 24 h treatment with 5 µM Yoda1 labelled for antibodies for PIEZO1 and microglial markers IBA1 CX3CR1, TMEM119, TREM2 and P2RY12. As a staining control only-secondary-antibody AF568 was used. **F** All mitochondrial parameters calculated from OCR values of mitostress assay in fig 1 and normalized to vehicle. N=5 in n=7. **G** Oxygen consumption rate (OCR) of iMGLs in mitostress test with 20 ng/ml LPS, vehicle and 5 µM Yoda1. N=3 in n=5. **H** Corresponding extracellular asidification (ECAR) curve. All data repeated in n=experiments with N biological replicates and n≥3 technical replicates in each experiment. Unpaired t-test, one-way ANOVA or two-way ANOVA. Significance ***p < 0.001, **p < 0.01, *p < 0.05. Data as mean ± SEM


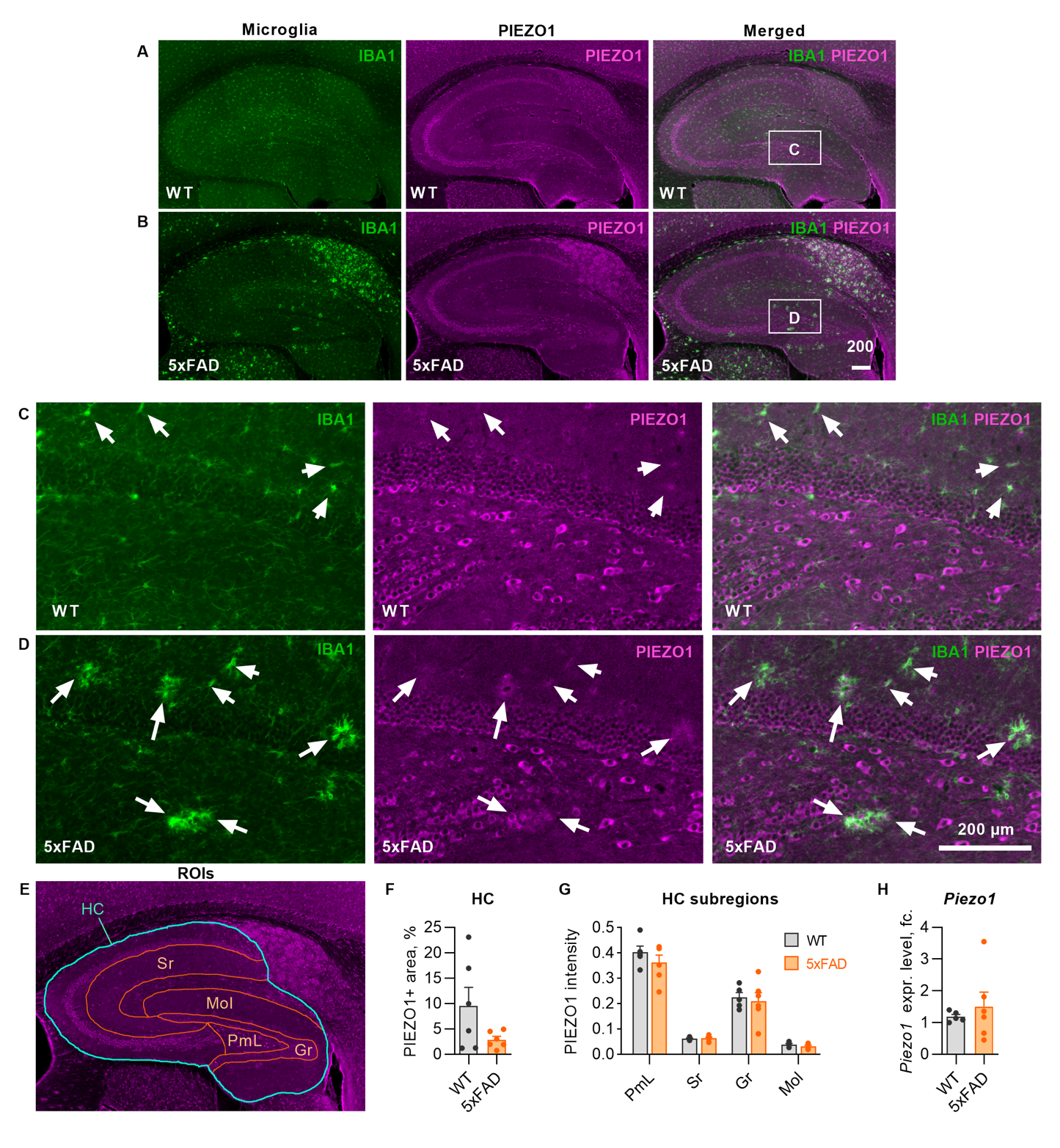
**Fig. S3. No differences in PIEZO1 expression in bulk brain tissue between WT and 5xFAD mice.** Representative immunofluorescence images of microglia (IBA1, green), PIEZO1 (magenta) and merged channels from sagittal hippocampal sections of 5-month-old **A** WT and **B** 5xFAD mice. **C-D** Magnifications with arrows indicating colocalization of IBA1 and PIEZO1 immunoreactivities Scale bars 200 µm. **E** Five regions of interest (ROIs) are delineated with to depict hippocampus (HC) (the outer most white line), stratum radiatum (Sr) and molecular layer (Mol), granular layer (Gr) and polymorph layer of dentate gyrus. **F** Quantification of the percentage of PIEZO1 immunoreactive area in the hippocampus, **G** and immunoreactivityof PIEZO1 staining within the subhippocampal regions. **H** Quantification of gene expression in a hemisphere. N=5-6 mice. Data as mean ± SEM.


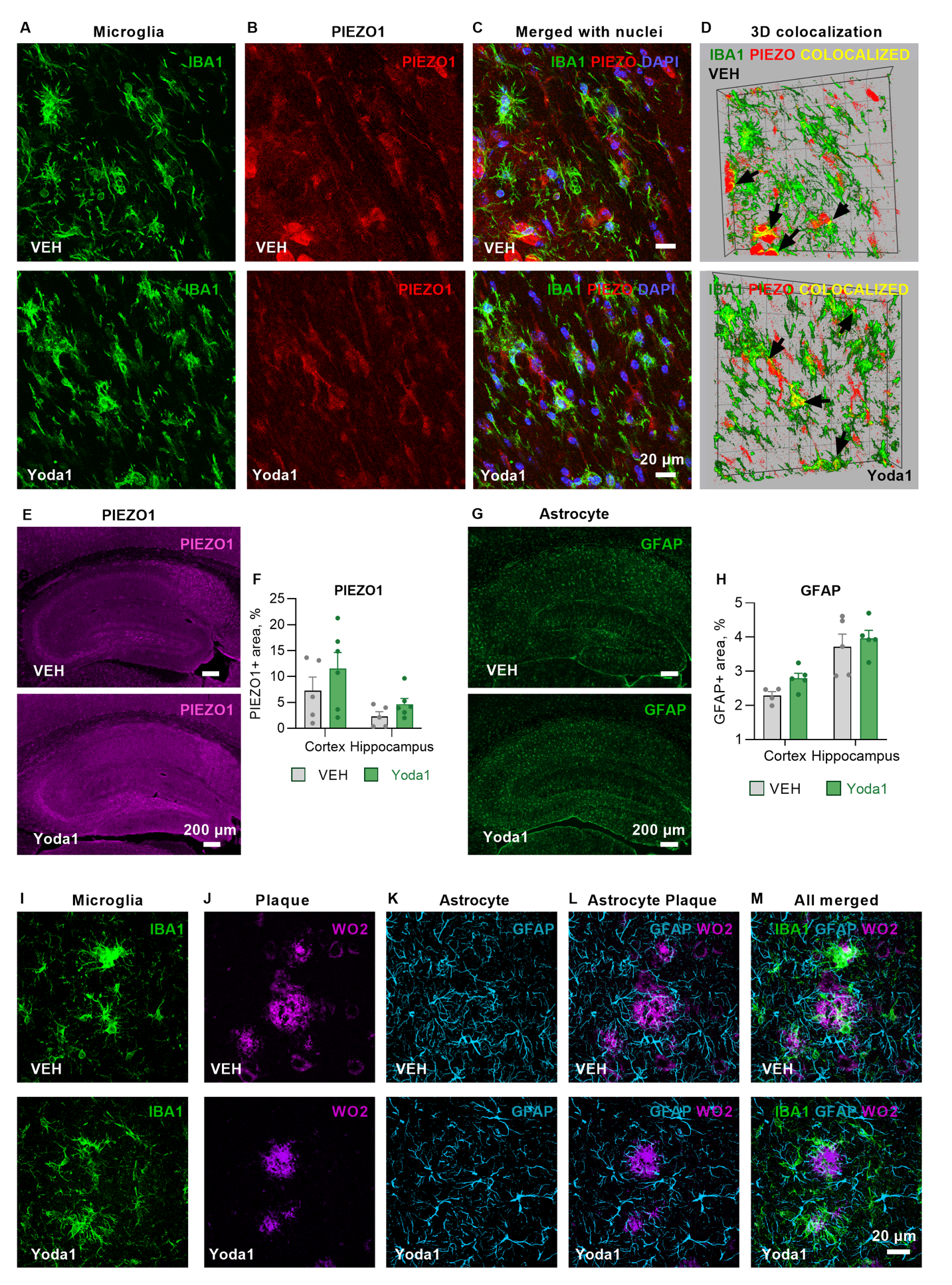
**Fig. S4. PIEZO1, microglia, astrocyte and Aβ plaque stainings in 5xFAD hippocampi.** Representative images of maximum intensity projections of confocal z-stacks with staining's for **A** microglia (IBA1, green) and **B** PIEZO1 (red) with **C** merged channels and **D** corresponding 3D reconstructions of the z-stacks showing co-localization of microglia and PIEZO1 (yellow) outside Aβ plaques in 5xFAD hippocampi. Scale bars 20 µm. **E** Representative immunofluorescence images of PIEZO1 (magenta) with **F** quantifications of immunoreactive area in hippocampus and cortex. Scale bar 200 µm. **G** Representative immunofluorescence images of astrocytes (GFAP, green) with **H** quantifications of immunoreactive area in hippocampus and cortex. Representative maximum intensity projections of confocal z-stack images of triple-immunostaining for **I** microglia (IBA1, green), **J** Aβ plaques (WO2, magenta), and **K** astrocytes (GFAP, green). **L** merged images showing no colocalization of GFAP and WO2 but **M** demonstrating clustering of IBA1 microglia around WO2 plaques. Scale bar 20 µm. N=5 VEH, N=6 Yoda1 mice. Unpaired t-test.


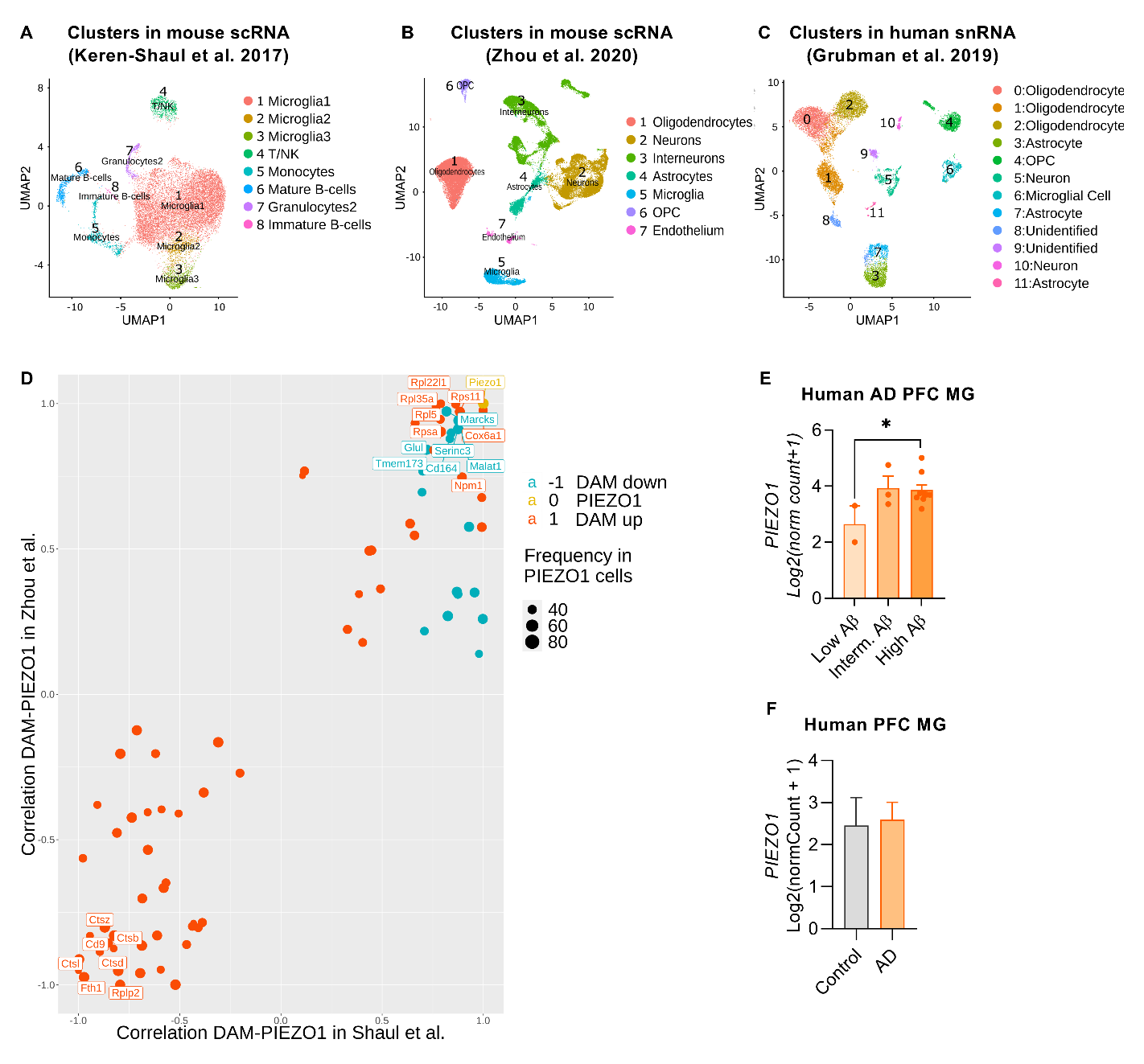
**Fig. S5. *PIEZO1* gene expression in published AD-related RNA datasets by our analysis.** UMAP visualizations of all cell clusters in **A** a 5xFAD mouse scRNA dataset [1], **B** Trem2^-/-^ 5xFAD snRNA dataset [2], and **C** human AD patient entorhinal snRNA dataset [3] as result of our clustering and annotation. **D** A correlation diagram between *Piezo1* and the DAM signature genes in microglial subpopulations in mouse datasets [1,2]. Size of dot represents gene frequency in *Piezo1*+ cells, color down or upregulated in DAM microglia, and location depends by the correlation of the gene with *Piezo1*. *PIEZO1* expression in human microglia isolated from postmortem prefrontal cortex (PFC; syn18485175) of AD patients **E** with different levels of Aβ burden and **F** bulk data compared to healthy controls. The samples with zero *PIEZO1* expression were excluded. Data obtained for E-F from http://www.brainrnaseq.org/. Unpaired t-test. Significance *p < 0.05. Data as mean ± SEM or min and max. **See also Tables S1-3.**

**
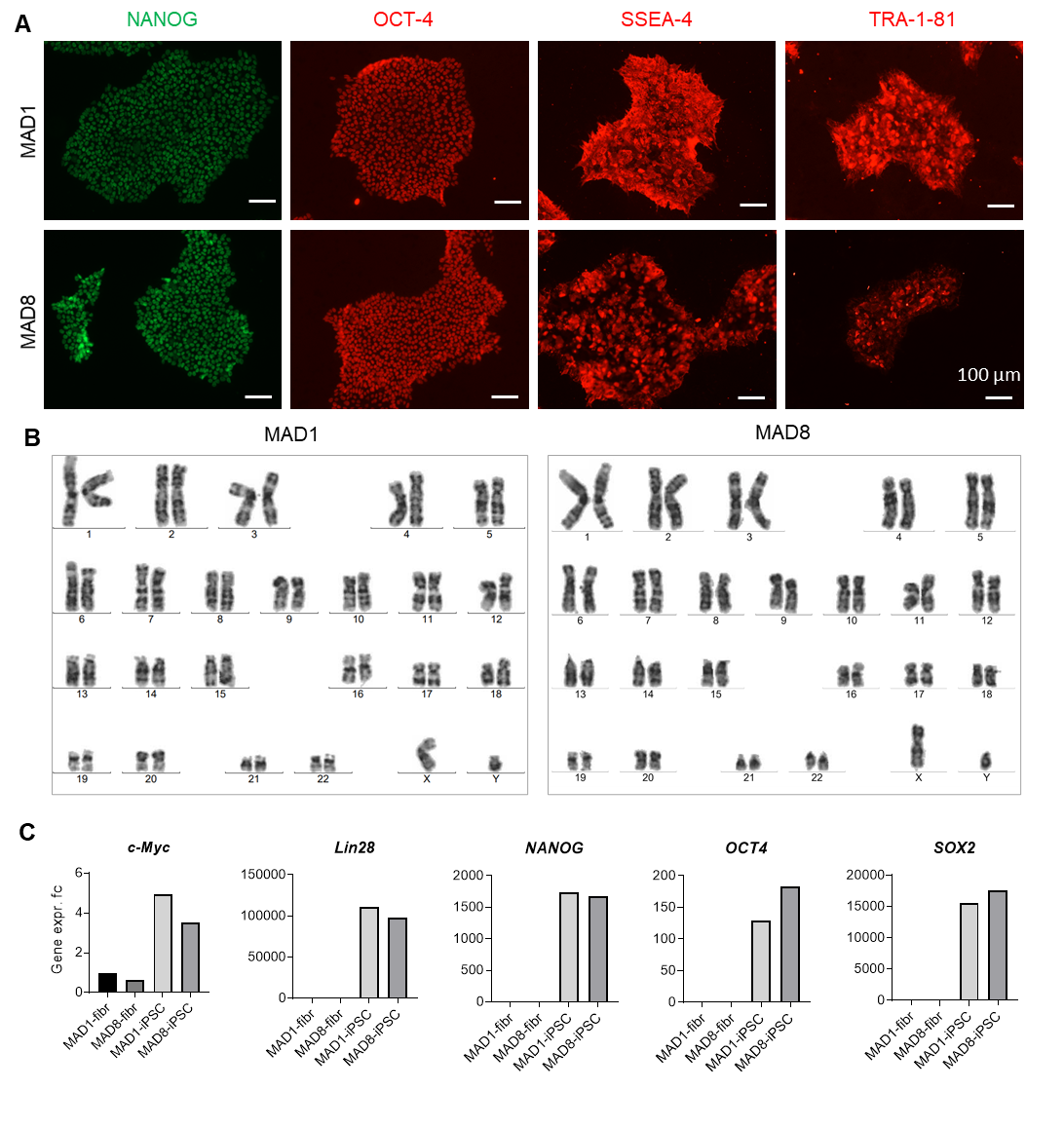
**

**Fig. S6. Characterization of MAD1 and MAD8 iPSC lines. A** Immunostaining images of the expression of pluripotency markers NANOG (green), OCT4, SSEA4 and TRA 1-81 (all red) in iPCS colonies. Scale bars 100 µm. **B** G-banding analysis of iPSCs performed by Ambar Lab (Anàlisis Mèdiques Barcelona) show normal 46 XY karyotypes. **C** RT-qPCR gene expression measurements of the pluripotency markers c-Myc (Hs00905030_m1), Lin28 (Hs00702808_s1), NANOG (Hs02387400_g1), OCT4 (Hs00742896_s1) and SOX2 (Hs01053049_s1) with fibroblasts (fibr) as negative controls. Showing mean of n=3 technical replicates.


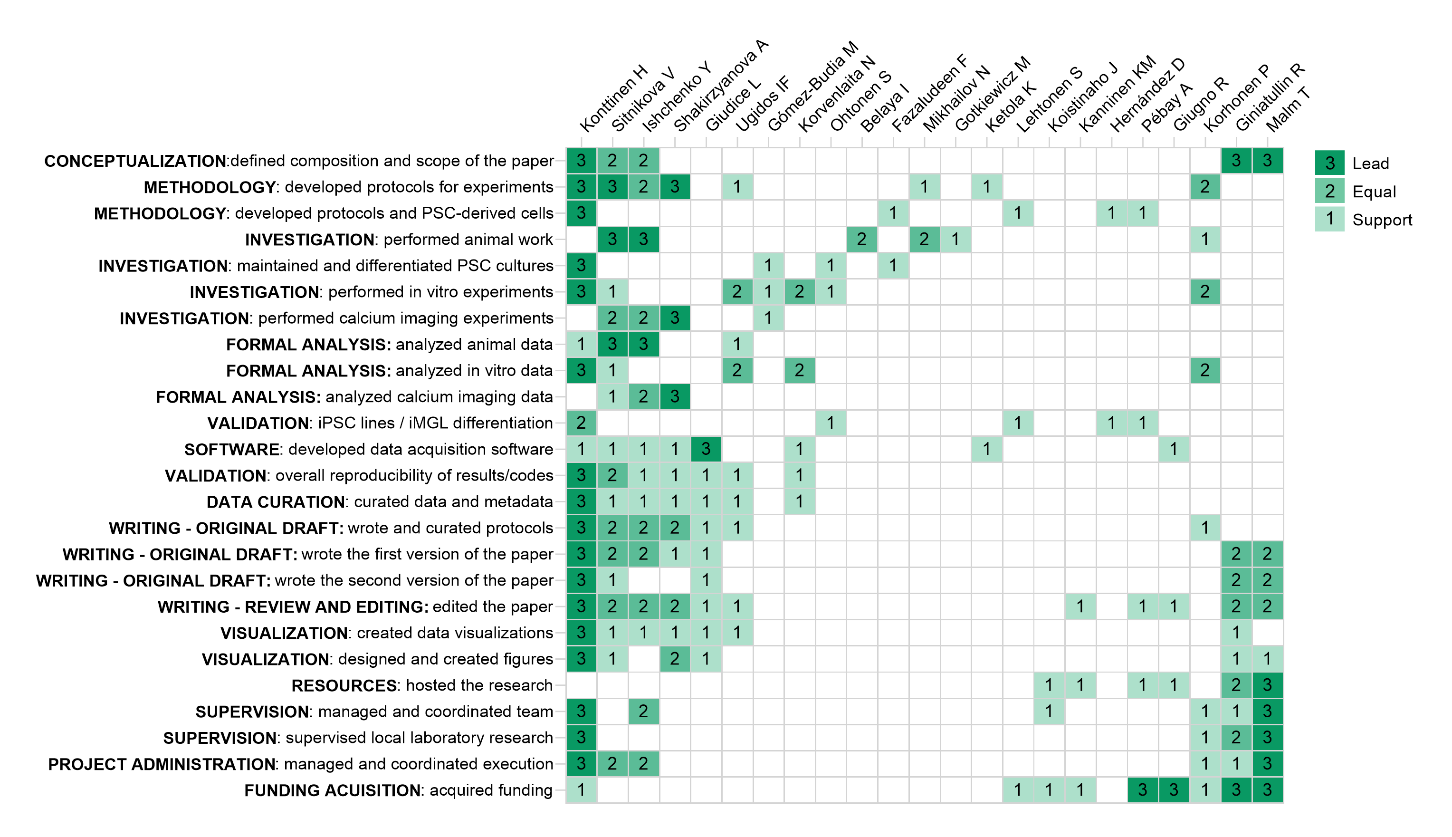
**Fig. S7. A diagram visualizing author contribution.** Based on the CRediT taxonomy [4]. For each type of contribution there are three levels indicated by color in the diagram: 1 support (light), 2 equal (medium), and 3 lead (dark).


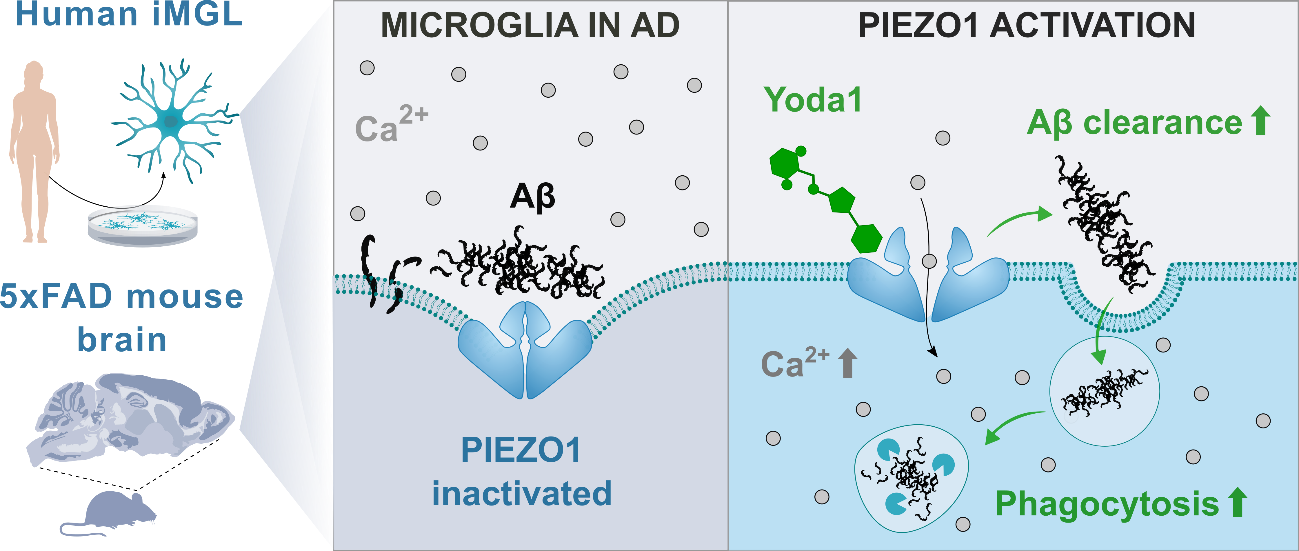


**Fig. S8. A graphical abstract summarizing the main finding of the paper.** Our data suggest that activating PIEZO1 mediated Ca^2+^ influx with a selective agonist Yoda1 triggers microglia to shift their function in such manner that leads to phagocytosis and lysosomal activation in human iMGL and murine microglia *in vitro* which could explain the observed clearance of Aβ *in vivo* in response to administration of Yoda1 to 5xFAD mice.

**Table S1.** A correlation data for *Piezo1* and the DAM signature genes in microglial subpopulations in Keren-Shaul et al. 2017 dataset (GSE98969 [1]).

| Gene ID | Cor.Shaul | Microglia1 | Microglia2 | Microglia3 (DAM) | DAMs labels | min_freq.Shaul |
| --- | --- | --- | --- | --- | --- | --- |
| *Cd164* | 0,84135765 | 0,992 | 0,974 | 0,965 | -1 | 0,965 |
| *Cox6a1* | 0,999093119 | 0,936 | 0,953 | 0,939 | 1 | 0,936 |
| *Glul* | 0,719356414 | 0,995 | 0,996 | 0,994 | -1 | 0,994 |
| *Malat1* | 0,87709347 | 0,997 | 0,996 | 0,965 | -1 | 0,965 |
| *Marcks* | 0,817983361 | 0,998 | 0,997 | 0,994 | -1 | 0,994 |
| *Npm1* | 0,894710871 | 0,949 | 0,897 | 0,985 | 1 | 0,897 |
| *Piezo1* | 1 | 1 | 0,998 | 0,997 | 0 | 0,997 |
| *Rpl22l1* | 0,862927676 | 0,835 | 0,725 | 0,939 | 1 | 0,725 |
| *Rpl35a* | 0,766646862 | 0,981 | 0,725 | 0,99 | 1 | 0,725 |
| *Rpl5* | 0,786998656 | 0,835 | 0,725 | 0,939 | 1 | 0,725 |
| *Rps11* | 0,883783624 | 0,98 | 0,984 | 0,985 | 1 | 0,98 |
| *Rpsa* | 0,790377515 | 0,975 | 0,984 | 0,977 | 1 | 0,975 |
| *Serinc3* | 0,875554683 | 0,999 | 0,998 | 0,994 | -1 | 0,994 |
| *Tmem173* | 0,70100146 | 0,983 | 0,989 | 0,939 | -1 | 0,939 |
| *Cd9* | -0,851741643 | 0,992 | 0,992 | 0,994 | 1 | 0,992 |
| *Ctsb* | -0,823464996 | 0,997 | 0,997 | 0,997 | 1 | 0,997 |
| *Ctsd* | -0,803587744 | 0,999 | 0,998 | 0,997 | 1 | 0,997 |
| *Ctsl* | -0,996532227 | 0,996 | 0,997 | 0,997 | 1 | 0,996 |
| *Ctsz* | -0,869983087 | 0,994 | 0,974 | 0,997 | 1 | 0,974 |
| *Fth1* | -0,972078623 | 0,996 | 0,996 | 0,997 | 1 | 0,996 |
| *Rplp2* | -0,793904002 | 0,982 | 0,984 | 0,985 | 1 | 0,982 |

DAM, disease associated microglia.

**Table S2.** A correlation data for *Piezo1* and the DAM signature genes in microglial subpopulations in Zhou et al. 2021 dataset (GSE140511 [2]).

| Gene ID | Cor.Zhou | 0 | 2 | 1 (DAM) | DAMs_labels.Zhou | min_freq.Zhou |
| --- | --- | --- | --- | --- | --- | --- |
| *Cd164* | 0,899358766 | 0,93563 | 0,90831 | 0,74772 | -1 | 0,74772 |
| *Cox6a1* | 0,97677366 | 0,87632 | 0,75998 | 0,86958 | 1 | 0,75998 |
| *Glul* | 0,841592869 | 0,98637 | 0,98273 | 0,95384 | -1 | 0,95384 |
| *Malat1* | 0,94186021 | 0,99995 | 0,99995 | 0,99995 | -1 | 0,99995 |
| *Marcks* | 0,97312609 | 0,99286 | 0,90831 | 0,9781 | -1 | 0,90831 |
| *Npm1* | 0,746656312 | 0,85851 | 0,85309 | 0,88715 | 1 | 0,85309 |
| *Piezo1* | 1 | 0,99995 | 0,99995 | 0,99995 | 0 | 0,99995 |
| *Rpl22l1* | 0,998392023 | 0,90536 | 0,75998 | 0,90364 | 1 | 0,75998 |
| *Rpl35a* | 0,990164613 | 0,82883 | 0,85309 | 0,90364 | 1 | 0,82883 |
| *Rpl5* | 0,946034325 | 0,79143 | 0,90831 | 0,84561 | 1 | 0,79143 |
| *Rps11* | 0,972373749 | 0,90536 | 0,9405 | 0,94114 | 1 | 0,90536 |
| *Rpsa* | 0,903238077 | 0,91614 | 0,98273 | 0,96333 | 1 | 0,91614 |
| *Serinc3* | 0,912671379 | 0,99286 | 0,9405 | 0,98715 | -1 | 0,9405 |
| *Tmem173* | 0,767940761 | 0,93956 | 0,75998 | 0,78813 | -1 | 0,75998 |
| *Cd9* | -0,855908668 | 0,98883 | 0,75998 | 0,99803 | 1 | 0,75998 |
| *Ctsb* | -0,829803352 | 0,99414 | 0,99641 | 0,99916 | 1 | 0,99414 |
| *Ctsd* | -0,950962476 | 0,99646 | 0,9875 | 0,99956 | 1 | 0,9875 |
| *Ctsl* | -0,911484438 | 0,9719 | 0,9405 | 0,99183 | 1 | 0,9405 |
| *Ctsz* | -0,801830694 | 0,9809 | 0,98273 | 0,99724 | 1 | 0,9809 |
| *Fth1* | -0,973093154 | 0,9843 | 0,99729 | 0,98878 | 1 | 0,9843 |
| *Rplp2* | -0,998825952 | 0,89256 | 0,96043 | 0,90364 | 1 | 0,89256 |

DAM, disease associated microglia.

**Table S3.** Differentially expressed genes (DEGs) specific for m1-subcluster in snRNA Grubman et al. 2019 dataset (GSE138852, [3]). # indicates the eight DEGs expressed at highest level in m1 and *AD GWAS genes presented by Grubman et al. 2019.

| Gene ID | p_val | avg_logFC | pct.1 | pct.2 | p_val_adj | # | * |
| --- | --- | --- | --- | --- | --- | --- | --- |
| *LINGO1* | 6.12E-54 | 2.105339101 | 0.879 | 0.374 | 5.83971E-50 | # |  |
| *MT-ND4* | 8.14E-18 | 1.745256134 | 0.561 | 0.15 | 7.77697E-14 | # |  |
| *MT-ND3* | 5.7E-12 | 1.690972913 | 0.379 | 0.048 | 5.44469E-08 |  |  |
| *BOK* | 1.3E-10 | 1.597021176 | 0.242 | 0.012 | 1.2433E-06 |  |  |
| *FCGBP* | 7.95E-10 | 1.583308972 | 0.318 | 0.051 | 7.59101E-06 |  |  |
| *CRYAB* | 6.71E-11 | 1.567980192 | 0.394 | 0.147 | 6.41161E-07 |  |  |
| *SNX6* | 1.44E-09 | 1.497222753 | 0.348 | 0.09 | 1.37798E-05 |  |  |
| *RPS28* | 1.69E-13 | 1.494904224 | 0.485 | 0.135 | 1.61514E-09 |  |  |
| *HSPA5* | 4E-08 | 1.490987216 | 0.258 | 0.033 | 0.000381564 |  |  |
| *MT-CO2* | 1.99E-11 | 1.475309993 | 0.47 | 0.138 | 1.90464E-07 | # |  |
| *GFAP* | 2.07E-08 | 1.433313829 | 0.288 | 0.051 | 0.000198083 |  |  |
| *MT-ND2* | 3.78E-08 | 1.432516142 | 0.333 | 0.078 | 0.000361407 | # |  |
| *NDRG1* | 2.05E-07 | 1.400422926 | 0.212 | 0.021 | 0.001960356 |  |  |
| *HSP90B1* | 3.48E-07 | 1.394687521 | 0.318 | 0.078 | 0.003325934 |  |  |
| *SPP1* | 5.48E-12 | 1.349364779 | 0.773 | 0.455 | 5.23015E-08 |  |  |
| *MT-ATP6* | 2.97E-08 | 1.338138732 | 0.318 | 0.075 | 0.000283221 | # |  |
| *MT-CO3* | 1.18E-11 | 1.311707022 | 0.53 | 0.129 | 1.12864E-07 | # |  |
| *MT-CYB* | 1.59E-08 | 1.263223432 | 0.394 | 0.105 | 0.000152088 | # |  |
| *PLEKHA6* | 4.56E-06 | 1.235822595 | 0.212 | 0.042 | 0.043528261 |  |  |
| *DNAJB2* | 3.31E-06 | 1.230809051 | 0.242 | 0.054 | 0.031584378 |  |  |
| *LINC00486* | 1.22E-39 | 1.204267915 | 1 | 0.979 | 1.16508E-35 |  |  |
| *APOC1* | 1.31E-06 | 1.177523416 | 0.288 | 0.099 | 0.01249037 |  | * |
| *RPL35* | 6.29E-07 | 1.102400971 | 0.394 | 0.144 | 0.006008747 |  |  |
| *VWA1* | 3.94E-06 | 1.101675686 | 0.152 | 0.009 | 0.037653863 |  |  |
| *ARMC9* | 5.39E-09 | 1.088684428 | 0.515 | 0.231 | 5.14294E-05 |  |  |
| *HSPA1A* | 1.16E-08 | 1.071470619 | 0.545 | 0.189 | 0.000110632 |  |  |
| *KANSL1L* | 4.29E-06 | 1.067163753 | 0.258 | 0.078 | 0.040951065 |  |  |
| *DPYD* | 6.98E-07 | 1.03478417 | 0.5 | 0.266 | 0.006662666 |  |  |
| *HSP90AA1* | 3.51E-07 | 1.032117928 | 0.439 | 0.141 | 0.003354925 |  |  |
| *RPL37A* | 2.23E-06 | 1.000686264 | 0.303 | 0.111 | 0.021285833 |  |  |
| *RPS19* | 2.54E-07 | 0.971602862 | 0.53 | 0.341 | 0.002423795 |  |  |
| *C1QC* | 6.19E-08 | 0.899225936 | 0.455 | 0.263 | 0.000591165 |  |  |
| *FMN1* | 9.03E-08 | 0.898793765 | 0.5 | 0.251 | 0.000862091 |  |  |
| *ACTB* | 6.81E-07 | 0.870135708 | 0.53 | 0.311 | 0.006503316 |  |  |
| *SERF2* | 1.72E-07 | 0.869088241 | 0.333 | 0.186 | 0.001645651 |  |  |
| *APOE* | 8.72E-08 | 0.822610932 | 0.697 | 0.533 | 0.000832737 |  | * |
| *RFWD2* | 4.32E-06 | 0.329535712 | 0.227 | 0.249 | 0.041250532 |  |  |
| *UBE2E2* | 1.49E-07 | -0.265467932 | 0.364 | 0.683 | 0.001421866 |  |  |
| *KCNMA1* | 2.59E-07 | -0.37860586 | 0.167 | 0.428 | 0.002471759 |  |  |
| *AOAH* | 2.31E-07 | -0.458036774 | 0.197 | 0.494 | 0.00220782 |  |  |
| *HS3ST4* | 3.71E-06 | -0.490835876 | 0.379 | 0.701 | 0.035405419 |  |  |
| *MEF2A* | 1.27E-08 | -0.537339438 | 0.545 | 0.874 | 0.000120936 |  |  |
| *TMEM117* | 1.29E-06 | -0.53980123 | 0.015 | 0.156 | 0.012358666 |  |  |
| *ANKRD44* | 2.35E-09 | -0.582183292 | 0.318 | 0.722 | 2.24312E-05 |  |  |
| *MEF2C* | 7.89E-08 | -0.593242864 | 0.576 | 0.88 | 0.000753268 |  | * |
| *C10orf11* | 1.2E-14 | -0.704657839 | 0.606 | 0.958 | 1.14534E-10 |  |  |
| *MALAT1* | 1.51E-39 | -0.719913013 | 1 | 1 | 1.44389E-35 |  |  |
| *FOXN3* | 8.51E-09 | -0.727831377 | 0.364 | 0.757 | 8.12227E-05 |  | * |
| *DOCK4* | 2.11E-09 | -0.757036602 | 0.758 | 0.949 | 2.01274E-05 |  |  |
| *ST6GAL1* | 1.54E-09 | -0.876638131 | 0.439 | 0.817 | 1.46945E-05 |  | * |
| *CAMK1D* | 2.54E-06 | -1.093782153 | 0.061 | 0.335 | 0.024263186 |  |  |
| *LPAR6* | 6.78E-09 | -1.109260972 | 0.227 | 0.629 | 6.47025E-05 |  |  |
| *USP53* | 1.34E-06 | -1.119665536 | 0.045 | 0.311 | 0.012819522 |  |  |
| *USP39* | 7.13E-07 | -1.161382456 | 0.076 | 0.38 | 0.00681296 |  |  |
| *ZFP36L2* | 5.21E-07 | -1.171247383 | 0.045 | 0.323 | 0.004977081 |  |  |
| *A2M* | 1.69E-06 | -1.173381626 | 0.182 | 0.497 | 0.016146244 |  |  |
| *SYNDIG1* | 7.3E-09 | -1.259017784 | 0.167 | 0.56 | 6.96651E-05 |  |  |
| *CD86* | 3.4E-06 | -1.305477311 | 0.076 | 0.356 | 0.032499009 |  |  |
| *BTG2* | 2.53E-06 | -1.323070172 | 0 | 0.177 | 0.024127191 |  |  |
| *RP11-624C23.1* | 5.72E-16 | -1.337719226 | 0.197 | 0.74 | 5.46382E-12 |  |  |
| *PRDM11* | 2.67E-07 | -1.363609996 | 0 | 0.207 | 0.002552475 |  |  |
| *APBA1* | 1.07E-07 | -1.379359477 | 0 | 0.219 | 0.001020117 |  |  |
| *FRMD4A* | 1.03E-22 | -1.382930811 | 0.439 | 0.919 | 9.87037E-19 |  | * |
| *OLR1* | 9.56E-07 | -1.405655026 | 0.106 | 0.404 | 0.009130846 |  |  |
| *KCNQ1* | 2.64E-08 | -1.470846884 | 0 | 0.237 | 0.000252285 |  | * |
| *SDK1* | 1.15E-08 | -1.471588901 | 0.106 | 0.467 | 0.000109763 |  |  |
| *CSGALNACT1* | 4.54E-10 | -1.565128643 | 0.061 | 0.446 | 4.33123E-06 |  |  |
| *MCF2L2* | 1.74E-07 | -1.576547828 | 0.045 | 0.344 | 0.001657942 |  |  |
| *ST6GALNAC3* | 4.49E-13 | -1.578137257 | 0.106 | 0.581 | 4.2879E-09 |  |  |
| *RP5-1031D4.2* | 1.07E-07 | -1.608265176 | 0 | 0.219 | 0.001020117 |  |  |
| *RP11-480C22.1* | 2.09E-08 | -1.628153071 | 0 | 0.24 | 0.000199362 |  |  |
| *DUSP1* | 1.07E-07 | -1.758573775 | 0 | 0.219 | 0.001020117 |  |  |
| *SRGN* | 4.29E-10 | -2.034698722 | 0.076 | 0.458 | 4.09934E-06 |  |  |

**References**

1. Keren-Shaul H, Spinrad A, Weiner A, Matcovitch-Natan O, Dvir-Szternfeld R, Ulland TK, et al. A Unique Microglia Type Associated with Restricting Development of Alzheimer’s Disease. Cell. Cell Press; 2017;169:1276-1290.e17.

2. Zhou Y, Song WM, Andhey PS, Swain A, Levy T, Miller KR, et al. Human and mouse single-nucleus transcriptomics reveal TREM2-dependent and TREM2-independent cellular responses in Alzheimer’s disease. Nat Med; 2020;26:131–42.

3. Grubman A, Chew G, Ouyang JF, Sun G, Choo XY, McLean C, et al. A single-cell atlas of entorhinal cortex from individuals with Alzheimer’s disease reveals cell-type-specific gene expression regulation. Nat Neurosci. Nature Research; 2019;22:2087–97.

4. Brand A, Allen L, Altman M, Hlava M, Scott J. Beyond authorship: Attribution, contribution, collaboration, and credit. Learn Publ; 2015;28:151–5.
